# Long-Lasting Response to Lorlatinib in Patients with ALK-Driven Relapsed or Refractory Neuroblastoma Monitored with Circulating Tumor DNA Analysis

**DOI:** 10.1158/2767-9764.CRC-24-0338

**Published:** 2024-09-30

**Authors:** Torben Ek, Raghda R. Ibrahim, Hartmut Vogt, Kleopatra Georgantzi, Catarina Träger, Jennie Gaarder, Anna Djos, Ida Rahmqvist, Elisabeth Mellström, Fani Pujol-Calderón, Christoffer Vannas, Lina Hansson, Henrik Fagman, Diana Treis, Susanne Fransson, Tobias Österlund, Tzu-Po Chuang, Bronte Manouk Verhoeven, Anders Ståhlberg, Ruth H. Palmer, Bengt Hallberg, Tommy Martinsson, Per Kogner, Martin Dalin

**Affiliations:** 1 Children’s Cancer Centre, Queen Silvia Children’s Hospital, Sahlgrenska University Hospital, Region Västra Götaland, Gothenburg, Sweden.; 2 Department of Pediatrics, Sahlgrenska Center for Cancer Research, Institute of Clinical Sciences, Sahlgrenska Academy at University of Gothenburg, Gothenburg, Sweden.; 3 Wallenberg Centre for Molecular and Translational Medicine, University of Gothenburg, Gothenburg, Sweden.; 4 Department of Biomedical and Clinical Sciences, Crown Princess Victoria Children’s Hospital, and Division of Children’s and Women’s Health, Linköping University, Linköping, Sweden.; 5 Department of Pediatric Hematology and Oncology, Karolinska University Hospital, Stockholm, Sweden.; 6 Childhood Cancer Research Unit, Women’s and Children’s Health, Karolinska Institutet, Stockholm, Sweden.; 7 Department of Pediatric Hematology and Oncology, Academic Children’s Hospital, Uppsala, Sweden.; 8 Department of Women’s and Children’s Health, Uppsala University, Uppsala, Sweden.; 9 Department of Laboratory Medicine, Institute of Biomedicine, Sahlgrenska Academy at University of Gothenburg, Gothenburg, Sweden.; 10 Department of Clinical Genetics and Genomics, Sahlgrenska University Hospital, Region Västra Götaland, Gothenburg, Sweden.; 11 Department of Laboratory Medicine, Sahlgrenska Center for Cancer Research, Institute of Biomedicine, Sahlgrenska Academy at University of Gothenburg, Gothenburg, Sweden.; 12 Department of Oncology, Sahlgrenska University Hospital, Region Västra Götaland, Gothenburg, Sweden.; 13 Department of Clinical Pathology, Sahlgrenska University Hospital, Region Västra Götaland, Gothenburg, Sweden.; 14 Department of Medical Biochemistry and Cell Biology, Institute of Biomedicine, Sahlgrenska Academy at University of Gothenburg, Gothenburg, Sweden.

## Abstract

Patients with anaplastic lymphoma kinase (ALK)–driven neuroblastoma may respond to tyrosine kinase inhibitors, but resistance to treatment occurs and methods currently used for detection of residual disease have limited sensitivity. Here, we present a national unselected cohort of five patients with relapsed or refractory ALK-driven neuroblastoma treated with lorlatinib as monotherapy and test the potential of targeted circulating tumor DNA (ctDNA) analysis as a guide for treatment decisions in these patients. We developed a sequencing panel for ultrasensitive detection of *ALK* mutations associated with neuroblastoma or resistance to tyrosine kinase inhibitors and used it for ctDNA analysis in 83 plasma samples collected longitudinally from the four patients who harbored somatic *ALK* mutations. All four patients with *ALK* p.R1275Q experienced major responses and were alive 35 to 61 months after starting lorlatinib. A fifth patient with *ALK* p.F1174L initially had a partial response but relapsed after 10 months of treatment. In all cases, ctDNA was detected at the start of lorlatinib single-agent treatment and declined gradually, correlating with clinical responses. In the two patients exhibiting relapse, ctDNA increased 9 and 3 months, respectively, before clinical detection of disease progression. In one patient harboring *HRAS* p.Q61L in the relapsed tumor, retrospective ctDNA analysis showed that the mutation appeared *de novo* after 8 months of lorlatinib treatment. We conclude that some patients with relapsed or refractory high-risk neuroblastoma show durable responses to lorlatinib as monotherapy, and targeted ctDNA analysis is effective for evaluation of treatment and early detection of relapse in ALK-driven neuroblastoma.

**Significance:**

We present five patients with ALK-driven relapsed or refractory neuroblastoma treated with lorlatinib as monotherapy. All patients responded to treatment, and four of them were alive after 3 to 5 years of follow-up. We performed longitudinal ctDNA analysis with ultra-deep sequencing of the *ALK* tyrosine kinase domain. We conclude that ctDNA analysis may guide treatment decisions in ALK-driven neuroblastoma, also when the disease is undetectable using standard clinical methods.

## Introduction

Neuroblastoma is a heterogeneous disease that accounts for 7% of all childhood malignancies (1). Tumors are characterized by risk-defining structural genetic alterations, such as amplification of *MYCN* and chromosomal copy number gains or losses (2, 3). Risk-based intensified multimodal treatments have improved survival, but clinical outcome is still unsatisfactory in children with high-risk disease, and effective targeted therapy may be necessary to further improve clinical outcome (4, 5). Mutations in the anaplastic lymphoma kinase (*ALK*) receptor tyrosine kinase occur in ∼10% of the cases at primary diagnosis, most commonly at positions F1174, F1245, or R1275 within the tyrosine kinase domain (6–10). Genetic alterations of *ALK* are associated with unfavorable prognosis when present at diagnosis and are identified in 20% to 43% of patients with relapsed or refractory neuroblastoma (11–17). Because effective second-line treatment options have been lacking, oncogenic *ALK* mutations provide an attractive target for targeted therapy with tyrosine kinase inhibitors (TKI). Although complete responses were reported, first- and second-generation ALK inhibitors crizotinib and ceritinib have shown limited clinical effect in most cases (18–20). On the other hand, the third-generation ALK TKI lorlatinib is highly effective against a wide range of *ALK* mutant variants expressed in neuroblastoma (21–23). A recent phase 1 trial of lorlatinib for patients with ALK-driven refractory or relapsed neuroblastoma showed relatively high response rates (24). However, resistance to lorlatinib was commonly observed, mediated by a variety of mechanisms such as secondary *ALK* mutations and mitogen-activated protein kinase pathway mutations (25).

Regular and accurate evaluation of tumor burden is crucial to provide the optimal treatment during the different phases of disease in patients with ALK-driven relapsed or refractory neuroblastoma. Currently, evaluation comprises imaging modalities such as ^123^I-meta-iodobenzylguanidine (MIBG) that cause radiation exposure and often require general anesthesia in pediatric patients. Computed tomography or magnetic resonance imaging scans are not able to differentiate between active disease and differentiated tumor tissue, and residual MIBG positivity may occasionally persist in patients without progressive disease after the end of treatment (26). Catecholamine metabolites in urine and blood and neuron-specific enolase (NSE) and chromogranin A in blood are frequently used for diagnosis and monitoring but have limited sensitivity for detection of relapse (27).

Circulating tumor DNA (ctDNA) has proved useful for investigating the evolution of tumor heterogeneity and therapy resistance in neuroblastoma (25, 28–30). However, its clinical utility for longitudinal monitoring of tumor burden at low levels of disease has not been thoroughly investigated.

Here, we present results from a national approach of thorough biological evaluation of all neuroblastomas and joint clinicobiological assessment of children with relapsed or refractory neuroblastoma for early implementation of targeted therapy using the third generation ALK-inhibitor lorlatinib for ALK-driven tumors. We report a neuroblastoma-*ALK* (NB-ALK) sequencing panel that enables ultrasensitive and highly specific detection of *ALK*-mutational positive ctDNA in patients with neuroblastoma treated with ALK TKIs. We used this method for long-term longitudinal analysis of minimal residual disease, identifying emergence of relapse prior to current clinical methodology. We report long-lasting clinical responses in five patients treated with lorlatinib, four of which harbored somatic *ALK* variants that permitted longitudinal monitoring of tumor burden. Our results support the use of lorlatinib as monotherapy in ALK-driven neuroblastoma and suggest that ctDNA analysis using the NB-ALK sequencing panel is effective for evaluation of treatment response and early detection of relapse in these patients.

## Materials and Methods

### Patients

This study includes all known patients with *ALK*-mutant neuroblastoma treated with lorlatinib as monotherapy in Sweden during 2016 to 2024. The patients met the following criteria: (i) metastatic relapsed or refractory neuroblastoma, (ii) a confirmed activating *ALK* mutation, (iii) not included in a clinical trial with an ALK inhibitor, and (iv) clinical recommendation of lorlatinib treatment by the Swedish ALK group, comprising pediatric oncologists from all six Swedish childhood cancer centers, clinical geneticists, and preclinical researchers. Written informed consent was obtained from all patients and/or legal guardians before initiation of treatment. The study was approved by the regional ethical review board in Gothenburg (Ref. nos 655-17, 485-16, and 1369-09), with amendments approved by the regional ethical review board (Ref. nos T795-16 and T525-18) and the Swedish Ethical Review Authority (Ref. nos 2019-06285 and 2021-04895). The study was conducted in compliance with the Declaration of Helsinki.

### Lorlatinib treatment

Lorlatinib was provided through the Pfizer Compassionate Use drug access program (reg. no. 2021-02829) or for the adult patient, prescribed as off-label treatment (Lorviqua). Treatment was administered as oral tablets with a starting dose of 75 to 95 mg/m^2^. The dose was adjusted according to side effects in some patients and was varied between 50 and 100 mg/m^2^. Complete response was defined according to the revised International Neuroblastoma Response Criteria (31). Working criteria for cessation of lorlatinib treatment were defined as (i) Complete or partial response according to the International Neuroblastoma Response Criteria, (ii) negative biochemical tumor markers (catecholamine metabolites, NSE, or chromogranin), (iii) no detectable *ALK* mutations in cell-free DNA with the NB-ALK sequencing panel, and (iv) a treatment duration of at least 2.5 years. The decision to stop ALK TKI treatment was made after careful discussion in the Swedish ALK group, in agreement with the patients and their family.

### Tumor and germline DNA sequencing

Whole-genome sequencing (WGS) of tumor and normal DNA was performed prospectively as part of the clinical diagnostic procedure. Tumor DNA from fresh frozen tumor tissue and germline DNA from the cell fraction of a blood sample collected at the time of diagnosis were extracted using the DNeasy Blood & Tissue Kits (QIAGEN). The concentration was measured with Qubit (Thermo Fisher Scientific) and DNA integrity was assessed with TapeStation (Agilent Technologies). Sequencing libraries were generated using TruSeq DNA PCR-Free library preparation kit (Illumina) with 1 µg of DNA input. WGS was done at Clinical Genomics, SciLifeLab, using NovaSeq (Illumina) with an intended average sequencing depth of 90× for tumor and 30× for germline DNA. Mapping to the human reference genome hg19, removal of read duplicates, and variant calling was performed using the Sentieon suite of bioinformatics tools (Sentieon Inc.). Single-nucleotide variants with a tumor allele frequency of at least 10% and a read coverage of at least 10 that passed manual review using Integrative Genomics Viewer were considered valid. An in-house Python script that calculated the fraction of shared single-nucleotide polymorphisms was used to confirm a genetic match between tumor and germline DNA in each case.

Mutations detected by WGS were confirmed using the Sanger sequencing of *ALK* exons 21 to 25 as previously described (10). PCR products were visualized on 2% agarose gels using UV light and GelRed (Biotium). All fragments were sequenced from both forward and reverse directions and analyzed using SeqScape software v3.0 (Applied Biosystems).

### Copy number analysis

Tumor samples were subjected to whole-genome profiling with Affymetrix CytoScan HD SNP microarray (Thermo Fisher Scientific) as previously described (32). GeneChip Command Console Software version 5.0.0.368 (Thermo Fisher Scientific) was used for primary data analysis, while genomic profiles and amplicon boundaries were determined using Chromosome Analysis Suite v.3.3.0 (Thermo Fisher Scientific).

### Blood sampling and extraction of cell-free DNA

Blood samples were collected by clinical nurses at the patients’ regular hospitals, using an existing central venous catheter or peripheral vein puncture. Blood was collected in cf-DNA/cf-RNA Preservative Tubes (Cat. 63950, Norgen Biotech), and plasma was prepared within 7 days of collection by centrifugation for 20 minutes at 420 RCF (Heraeus Megafuge 8R, Thermo Fisher Scientific). Plasma was transferred to XLX 2000–2D Biobanking Tubes (LVL Technologies) using the Freedom EVO liquid handling robot (Tecan Life Sciences) and stored at −80°C until cell-free DNA (cfDNA) extraction. Plasma was then thawed in water at room temperature and centrifuged for 10 minutes at 16,000*g* using a 5804R Centrifuge (Eppendorf). cfDNA was extracted from 1.4 to 6.2 mL plasma using the QIAamp Circulating Nucleic Acid Kit (QIAGEN), according to the manufacturer’s instructions with an elution volume of 150 µL. The extracted cfDNA was analyzed with Qubit dsDNA HS Assay Kit (ThermoFisher Scientific) and concentrated using Vivacon 500 centrifugal units with a 30 kDa molecular weight cutoff (Sartorius) to a final volume of 10 to 14 µL. Inhibition test was done using a 74-bp *PDGFRA* quantitative PCR assay, with Roche Human Genomic DNA (Sigma-Aldrich) as the positive control, as described previously (33).

### The NB-ALK sequencing panel

To achieve deep and accurate sequencing of *ALK* mutations recurrently detected in neuroblastoma and mutations associated with resistance to ALK inhibitors, we developed the NB-ALK sequencing panel. We used simple, multiplexed, PCR-based barcoding of DNA for ultrasensitive mutation detection by next-generation sequencing (SiMSen-seq; ref. 34) that included unique molecular identifiers (UMI) to minimize polymerase-induced errors and quantification bias. Primers (Integrated DNA Technologies) were designed using Primer-BLAST (National Center for Biotechnology Information), with annealing temperatures of 60°C to 63°C and amplicon sizes between 75 and 105 bps. Target primers were tested by qRT-PCR using Roche Human Genomic DNA (Sigma-Aldrich), followed by a melting curve analysis using SYBR Green I detection chemistry. The resulting amplicon size was confirmed by QIAxcel Advanced System (QIAGEN). After validation of the target primers, universal SiMSen-seq sequences were added to each target primer. The SiMSen-seq assays were tested as singleplex and as multiplex reactions, as described previously (34).

### Detection of HRAS p.Q61L

In patient 5, cell-free DNA from 2 mL of plasma remaining after NB-ALK panel sequencing was analyzed to assess the *HRAS* Q61 locus. Primer sequences were 5′-GCC​CTC​CCC​GGT​GCG​CAT​GTA-3′ (forward) and 5′-GGA​GAC​GTG​CCT​GTT​GGA​CAT​C-3′ (reverse), which resulted in an amplicon size of 88 bps. Sequencing was performed with SiMSen-seq as described for the NB-ALK panel.

### ctDNA analysis

Library construction in SiMSen-seq was performed in two PCR steps: barcoding PCR and adapter PCR. Barcoding PCR was performed in 10 µL, containing 1× Platinum SuperFi buffer, 0.2 U Platinum SuperFi DNA polymerase (No. 12351010, Thermo Fisher Scientific), 0.2 mmol/L dNTP (Sigma-Aldrich), 40 nmol/L of each SiMSen-seq barcoding primer, 0.5 mol/L L-carnitine inner salt (Sigma-Aldrich), and up to 20 ng of cfDNA. The temperature profile was 98°C for 3 minutes followed by three cycles of amplification (98°C for 10 seconds, 62°C for 6 minutes, and 72°C for 30 seconds), 65°C for 15 minutes, and 95°C for 15 minutes. In the beginning of the 65°C step, 45 ng of protease (*Streptomyces griseus*, Sigma Aldrich) dissolved in 30 μL Tris-EDTA buffer pH 8.0 (Ambion, Thermo Fisher Scientific) was added to inactivate the DNA polymerase. The adapter PCR was performed in 60 µL, containing 400 nmol/L of each of the universal forward and reverse adapter primers (Integrated DNA Technologies), 1× Q5 Hot Start High-Fidelity Master Mix, and 15 µL of barcoding PCR product. The temperature profile was 98°C for 3 minutes followed by 27 amplification cycles (98°C for 10 seconds, ramping from 80°C for 1 second down to 72°C for 30 seconds and up 76, 0.2°C/second, 76°C for 30 seconds). The PCR product was purified using the Agencourt AMPure XP beads system (No. A63881, Beckman Coulter, Inc.) with a 1:1 sample-to-beads ratio. Libraries were analyzed with HS NGS Fragment Kit (No. DNF-474, Agilent Technologies) on a 5200 Fragment Analyzer (Agilent Technologies), according to the manufacturer’s instructions. Sequencing of the *ALK* library pool at a final concentration of 1.8 pM was performed with the MiniSeq System (Illumina) using High Output Reagent Kit (150 cycles) containing 10% PhiX and using single-end reads with 150-bp mode.

### Bioinformatical analysis

NB-ALK sequencing data were analyzed bioinformatically with UMIErrorCorrect as described previously (35). In brief, sequencing reads were aligned to the Human Build 38 reference genome, and reads were grouped into UMI families based on target DNA regions and UMI sequence. Next, error-corrected consensus reads were generated requiring at least three raw reads in each UMI family. Plasma samples with at least one consensus read showing the hotspot mutation detected in the tumor at the time of diagnosis were considered ctDNA positive. Other nonsynonymous variants with at least 1% allele frequency were manually reviewed for accuracy using Integrative Genomics Viewer version 2.16.2 (broadinstitute.org/igv).

### Clinical tumor markers

Standard neuroblastoma tumor markers were analyzed as part of the clinical routine at each patient’s hospital. Serum NSE was analyzed with electrochemiluminescence (ECL), serum chromogranin A with immunofluorescence (Time Resolved Amplified Cryptate Emission technology), plasma-methoxy norepinephrine with LC/MS-MS, and the urine catecholamine metabolites homovanillic acid, vanillylmandelic acid, and dopamine with mass spectrometry. Only tumor markers that were elevated at the start of lorlatinib treatment and followed longitudinally are presented for each patient.

### Data availability

The data generated with the NB-ALK sequencing panel are available in the National Center for Biotechnology Information Sequence Read Archive (BioProject accession number PRJNA1150364). Whole-genome sequencing data are not publicly available for patient privacy reasons. Additional data not included in the article are available on request.

## Results

### Patient characteristics

All five patients with relapsed or refractory *ALK* mutation-positive neuroblastoma who have received lorlatinib monotherapy in Sweden so far were included in the study (Table 1). The median age was 9.8 years at diagnosis and 11.2 years at initiation of lorlatinib treatment. All primary tumors were located abdominally, with metastases in at least two compartments. Four patients harbored an *ALK* p.R1275Q mutation (in the absence of *MYCN* amplification), and one had an *ALK* p.F1174L mutation in combination with *MYCN* amplification. All *ALK* mutations were somatic except in patient 3, who had a germline *ALK* p.R1275Q mutation (Supplementary Fig. S1). Segmental chromosomal alterations included a 1p deletion in patients 1 and 5, an 11q deletion in patient 2, and the gain of 17q or whole chromosome 17 in patients 1, 2, 4, and 5 (Supplementary Fig. S2; Supplementary Data S1). Patients were initially treated according to the SIOPEN high-risk neuroblastoma-1 protocol (ClinicalTrials.gov ID: NCT01704716) and were started on lorlatinib monotherapy due to refractory disease (*n* = 3) or metastatic relapse (*n* = 2). The time between the primary diagnosis and start of lorlatinib was 7 to 26 months (Table 2).

**Table 1:** Baseline information

Patient	Sex	Age^a^ (y, m)	Primary tumor site	Metastatic compartments	*ALK* mutation	Structural alterations		Treatment before lorlatinib
						MNA	Segmental alterations	
1	F	9, 8	Left adrenal	Pancreas, LN	p.R1275Q	−	1p del, 1q del, chr11 loss, chr17 gain	RC-Sx-RT-RA
2	F	10, 1	Left adrenal	BM, CNS, bone, LN	p.R1275Q	−	1q del, 2p gain, chr17 gain, 11q del	RC-2TVD-Sx-2TVD-HDC+AHCT-RT
3	M	0, 2	Bilateral adrenal	BM, liver, lung, bone, CNS, skin, LN	p.R1275Q germline	No data^b^		RC-TVD
4	F	18, 5	Retroperitoneal	Bone, distant LN	p.R1275Q	−	1q del, chr17 gain	RC-Sx-2TVD
5	M	2, 2	Right adrenal	BM, lung, liver, bone, distant LN	p.F1174L	+	1p del, 17q gain	RC-Sx-HDC+ASCT-RT-RA+aGD2

a At time of primary diagnosis.

b No data available because array-comparative genomic hybridization showed a flat profile.

Abbreviations: aGD2, anti-GD2 monoclonal antibody (dinutuximab); ASCT, autologous hematopoietic cell transplantation; BM, bone marrow; CNS, central nervous system; del, deletion; HDC, high-dose chemotherapy (busulfan and melphalan); LN, lymph nodes; MNA, *MYCN* amplification; RA, retinoic acid; RC, rapid COJEC; RT, radiotherapy; Segm., segmental; Sx, surgery; TVD, topotecan, vincristine, and doxorubicin.

**Table 2:** Lorlatinib treatment information

Patient	Time from diagnosis to lorlatinib (months)	Disease status at start of lorlatinib	Lorlatinib dose (mg/m^2^)	First response, months/best response, months	Time on lorlatinib/follow-up (months)
1	26	PD (met. relapse)	75–100–50	PR, 2.0/CR, 11.2	40.6/14.6
2	13	Refractory	75–45	CR, 6.2/CR, 6.2	31.5/9.6^a^
3	7	Refractory	50–90	PR, 5.7/CR, 10	35.4/25.5
4	8	Refractory	56	PR, 2.8/PR (met. CR), 5.0	35.3 (lorlatinib ongoing)
5	11	PD (met. relapse)	98	PR, 2.0/PR, 4.5	10.5^a^

a Relapse.

Abbreviations: CR, complete response; met, metastatic; PD, progressive disease; PR, partial response.

### Lasting responses to lorlatinib monotherapy in patients with ALK p.R1275Q

**Figure 1:**
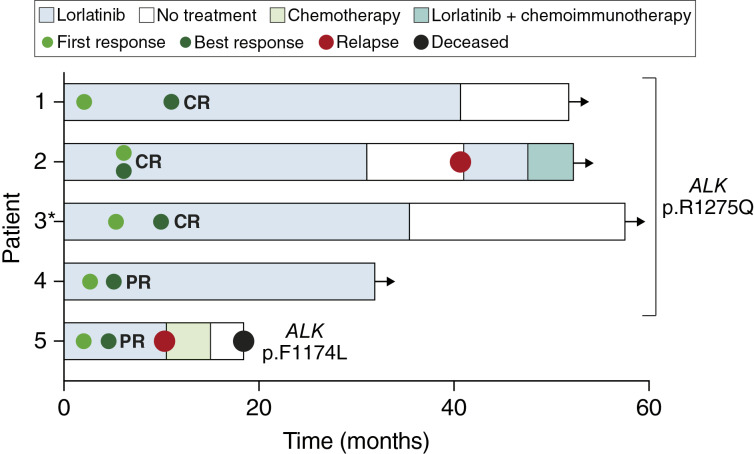
Clinical response to lorlatinib in patients with ALK-driven relapsed or refractory neuroblastoma. Black arrows denote ongoing treatment or follow-up. *Germline *ALK* variant. CR, complete response; PR partial response.

All five patients experienced reduced disease symptoms within days to weeks after starting lorlatinib. The median time to first radiologic response was 2.8 months, and the median time to best response was 6.2 months (Fig. 1). Three patients had a complete response and two exhibited partial responses. All four patients harboring an *ALK* p.R1275Q mutation were alive at the latest follow-up, on average 52 months (range 35–61 months) after starting lorlatinib. The patient with *ALK* p.F1174L and *MYCN* amplification had a metastatic relapse after 10 months of lorlatinib treatment and passed away 8 months later.

The most common side effects of lorlatinib were weight gain and grade 1 to 2 hypercholesterolemia, resulting in four patients being treated with statins. Also, three of the patients experienced grade 1 to 2 neurocognitive symptoms during the treatment (Supplementary Table S1). For a detailed summary of the clinical course of each patient, see Supplementary case information.

### The NB-ALK sequencing panel for sensitive detection of oncogenic ALK variants

**Figure 2:**
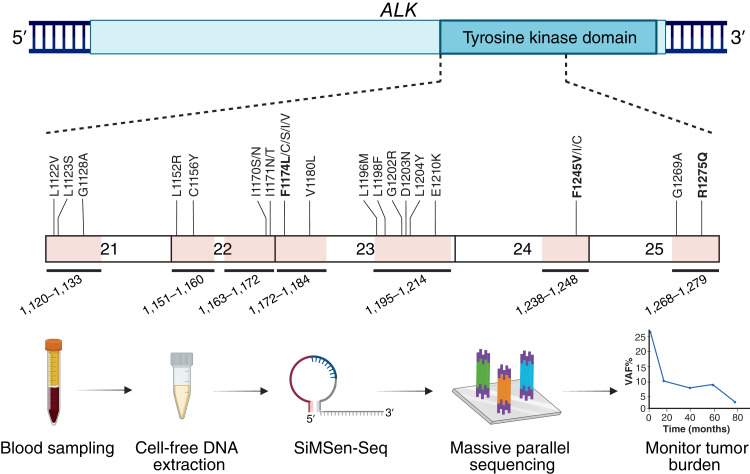
ctDNA analysis using the NB-ALK sequencing panel. Pink areas denote parts of the *ALK* gene included in the panel. Recurrent mutations previously detected in neuroblastoma or associated with resistance to TKIs that are covered by the panel are shown; neuroblastoma hotspot mutations are bold. Angled numbers denote the range of *ALK* amino acid positions covered by each of the seven assays.

The NB-ALK panel was designed to cover 20 *ALK* mutations previously described in neuroblastoma tumors or associated with TKI resistance (Supplementary Table S2; refs. 36–38). The panel includes seven assays in *ALK* exons 19 and 22 to 25 with amplicon sizes of 79 to 104 bps (Supplementary Table S3). The total targeted sequence is 351 bps, corresponding to approximately one-third of the *ALK* tyrosine kinase domain (Fig. 2).

To enable ultrasensitive mutation analysis, we applied SiMSen-seq, which labels each initial target DNA molecule with UMIs before amplification to minimize sequencing errors (34). In total, we analyzed 83 plasma samples from four patients (mean 21; range 13–31 samples/patient; the fifth patient’s germline *ALK* mutation precluded useful analysis) with a mean sampling period of 30 months (range 12–50 months). The average sequencing depth was ∼103,000 reads (Supplementary Fig. S3A). At the site of the oncogenic mutation, a mean of 5,180 original DNA molecules with different UMIs were sequenced at least three times each (Supplementary Fig. S3B). This corresponds to a variant allele frequency of 0.02% as the limit of detection. All sequencing results are found in Supplementary Data S2 (for all genomic positions covered by the NB-ALK sequencing panel) and Supplementary Data S3 (for a summary of the data at the hotspot mutation sites).

### ctDNA analysis enables sensitive evaluation of response to lorlatinib and early detection of relapse in patients with ALK p.R1275Q

**Figure 3:**
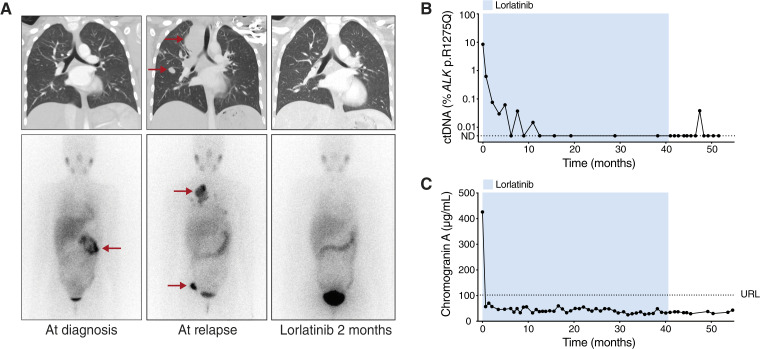
Lasting response to lorlatinib in a patient (patient 1) with relapsed neuroblastoma harboring *ALK* p.R1275Q. **A,** Chest computed tomography (top) and MIBG scans (bottom) at time of initial diagnosis, at relapse, and after 2 months of lorlatinib treatment. Red arrows denote tumors. **B,** Levels of ctDNA during and after lorlatinib treatment. ND, not detected. **C,** Chromogranin A. URL, upper reference limit (102 µg/L). NSE, urine VMA and urine HVA were within normal ranges at time of relapse and were therefore not further analyzed. HVA, homovanillic acid; VMA, vanillylmandelic acid.

All four patients with *ALK* p.1275Q experienced long-lasting responses to lorlatinib. Patient 1 presented with a stage M, *ALK* p.R1275Q-positive neuroblastoma at 9 years of age and had a metastatic relapse in the mediastinum, lungs, and hip bone 2 years after the initial diagnosis (Fig. 3A). After two cycles of irinotecan and temozolomide, the patient was transferred to lorlatinib monotherapy, which resulted in prompt relief of the symptoms. The levels of ctDNA decreased gradually over the first year and remained negative during the rest of the treatment, which lasted for 40 months (Fig. 3B). Eleven of the 12 plasma samples analyzed during follow-up were *ALK* mutant ctDNA negative, whereas one *ALK* p.R1275Q molecule was detected in one sample. However, this was followed by four negative time points, and the patient remains asymptomatic and free from detectable disease 14 months after ending lorlatinib treatment. The clinical tumor marker chromogranin A normalized after 2 weeks of lorlatinib treatment and has remained within the reference interval (Fig. 3C).

**Figure 4:**
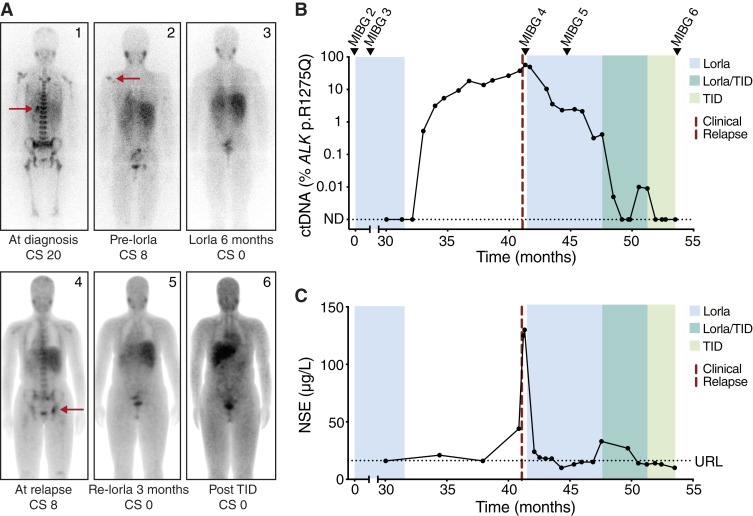
Detection of relapse with ctDNA analysis 9 months prior to clinical presentation in a patient (patient 2) with *ALK* p.R1275Q-positive neuroblastoma treated with lorlatinib. **A,** MIBG scans. **B,** Levels of ctDNA. **C,** NSE. CS, Curie score; ND, not detected; TID, temozolomide, irinotecan and dinutuximab; URL, upper reference limit (16.3 µg/L).

Patient 2 was diagnosed with high-risk neuroblastoma at 10 years of age (Fig. 4A). Because response evaluation after induction and consolidation therapy showed refractory disease, the patient was taken off protocol and treated with lorlatinib. This led to a complete response which persisted throughout 31 months of treatment. Ten months after cessation of lorlatinib treatment, the patient presented with a disseminated relapse in bone and bone marrow (SIOPEN score 8). Retrospective ctDNA analysis revealed circulating *ALK* p.R1275Q at increasing levels starting 9 months prior to clinical presentation of relapse (Fig. 4B). Of note, the levels of the clinical biomarker NSE were not increased until clinical detection of relapse 10 months after the cessation of lorlatinib therapy, highlighting the clinical superiority of the ALK-NB sequencing panel in early detection of relapse (Fig. 4C). The patient responded well to reintroduction of lorlatinib, correlating with declining levels of ctDNA. After 6 months of treatment with lorlatinib as monotherapy, *ALK* mutant ctDNA was still detectable. This led to intensified treatment with temozolomide and irinotecan for 5 days combined with dinutuximab for 7 days in 10 cycles given with 21 days interval. Lorlatinib treatment was discontinued after five treatment cycles. At the end of this treatment, the patient had no evidence of disease and *ALK* mutant ctDNA levels were negative in the last four samples.

Patient 3 presented with widely metastasized high-risk neuroblastoma at 2 months of age and had resistant disease with continued bone marrow activity and leptomeningeal carcinomatosis after neoadjuvant chemotherapy. Identification of a germline *ALK* p.1275Q mutation resulted in subsequent lorlatinib monotherapy, which resulted in a complete response lasting for 35 months during treatment and for an additional 25 months of follow-up time without treatment. Because it was a germline mutation and therefore expected to be detectable in the patient’s blood at all time points regardless of tumor burden, this patient was excluded from the *ALK* mutation ctDNA analysis.

**Figure 5:**
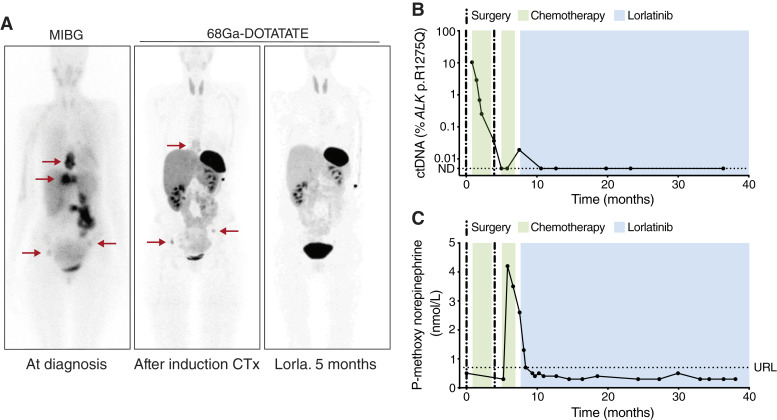
Long-term response to lorlatinib in a patient (patient 4) with refractory *ALK* p.R1275Q-positive neuroblastoma. **A,** MIBG or ^68^Ga-DOTATATE scans at the time of diagnosis, after induction of chemotherapy, and after 5 months of lorlatinib treatment. Red arrows denote tumors. **B,** Levels of ctDNA. **C,** Plasma methoxy noradrenaline. ND, not detected; URL, upper reference limit (0.7 nmol/L).

Patient 4 was diagnosed with neuroblastoma at 18 years of age and underwent acute tumor resection due to spinal cord compression. Rapid COJEC chemotherapy resulted in stable disease, and reevaluation after debulking surgery and two additional cycles of chemotherapy showed tumor progression. The patient was then taken off protocol and treated with lorlatinib as monotherapy, which resulted in a complete response at metastatic sites together with a partial response in the primary tumor (Fig. 5A). A cystic tumor remained, with a complete resolution on ^68^Ga-DOTATOC scan. The patient remains asymptomatic after 35 months of lorlatinib treatment. *ALK* mutant–positive ctDNA gradually reduced during neoadjuvant chemotherapy and was negative after the second surgery. Low levels detected on initiation of lorlatinib treatment were followed by undetectable levels throughout the remaining treatment course (Fig. 5B). The plasma level of methoxy norepinephrine was not elevated at diagnosis but increased during adjuvant chemotherapy. Levels normalized during the initial period of lorlatinib treatment have subsequently remained within the reference interval (Fig. 5C).

### Identification of a *de novoHRAS* p.Q61L resistance mutation in response to lorlatinib treatment

**Figure 6:**
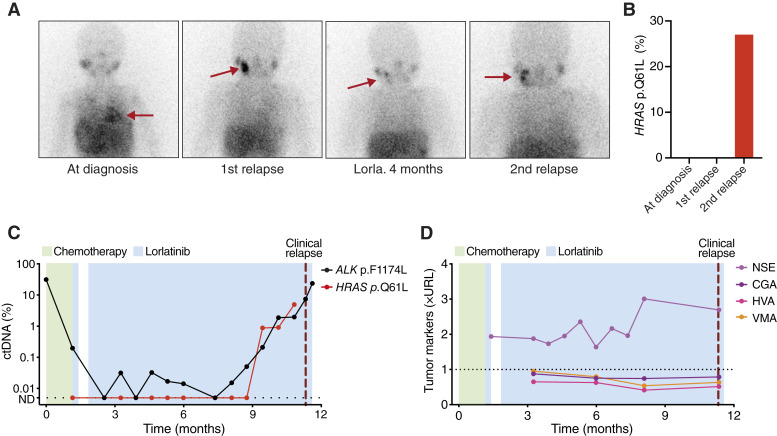
Early detection of relapse and emergence of an *HRAS* p.Q61L mutation during lorlatinib treatment in a patient (patient 5) with *ALK* p.F1174L-positive neuroblastoma. **A,** MIBG scans at initial diagnosis, at the first relapse, after 4 months of lorlatinib (partial response), and at second relapse after 10.5 months of lorlatinib treatment. **B,** Allele frequency of *HRAS* p.Q61L in tumor tissue at different time points. **C,** Levels of ctDNA. **D,** Clinical tumor markers, values are normalized to the upper reference limit (URL) for each marker. CGA, chromogranin A; Lorla, lorlatinib; ND, not detected.

Patient 5 was diagnosed at 22 months of age with neuroblastoma in the adrenal gland with metastatic sites in the lungs, liver, mandible, and bone marrow. Genetic analysis identified an *ALK* p.F1174L mutation together with amplification of *MYCN*. During maintenance treatment, a relapse in the mandible was detected, and after two cycles of chemotherapy, the patient was switched to lorlatinib as monotherapy with an initial partial response. MIBG scans showed a second mandibular relapse after 10.5 months of lorlatinib (Fig. 6A). WGS of DNA from the relapsed tumor identified an *HRAS* p.Q61L mutation. To investigate the occurrence of this mutation in the patients’ samples over time, we developed an ultrasensitive *HRAS* Q61 sequencing assay, using similar methods as for the NB-ALK sequencing panel. *HRAS* p.Q61L was not present in tumor DNA at diagnosis or at first relapse but was detected with an allele frequency of 27% in the second relapse occurring during lorlatinib treatment (Fig. 6B). In line with this, the mutation was undetectable in cfDNA during the first phase of lorlatinib therapy and became positive at stepwise increasing levels starting 8 months into the treatment (Fig. 6C).

In contrast to the previous patients, although circulating *ALK* p.F1174L levels decreased, they were not consistently undetectable in response to lorlatinib treatment. Furthermore, increasing levels of *ALK* p.F1174L-positive ctDNA were detected 3 months before the relapse was diagnosed (Fig. 6C). Of note, clinically used tumor markers were not able to detect the relapse. NSE was mildly elevated throughout the lorlatinib treatment and did not increase further at the time of relapse, whereas chromogranin A, homovanillic acid, and vanillylmandelic acid remained within normal ranges at all time points (Fig. 6D).

### No secondary ALK mutations were detected in circulating DNA or tumor tissue

Mutations in the *ALK* tyrosine kinase domain commonly arise in tumors resistant to ALK TKI treatment and have been reported in patients with neuroblastoma treated with ALK inhibitors (25). Therefore, we used the NB-ALK sequencing panel data to analyze all 83 cell-free DNA samples for secondary *ALK* mutations as a relapse mechanism. Potential secondary mutations were defined as any nonsynonymous variant with an allele frequency in cfDNA of at least 1%. No mutations meeting these criteria were identified in the plasma samples from the four patients analyzed.

We also used the NB-ALK sequencing panel for analysis of tissue DNA from 10 tumor samples; four primary tumor biopsies and six samples acquired at relapse or disease progression. The oncogenic *ALK* mutations detected by WGS as part of the clinical workup (p.R1275Q or p.F1174L) were confirmed in all tumors with an allele frequency of 16% to 70% (Supplementary Fig. S4). In patient 2, we also detected *ALK* p.R1275Q in both samples of bilateral bone marrow biopsies at the time of the second relapse. However, no potential resistance mutations in the *ALK* tyrosine kinase domain were detected in the 12 tissue samples.

## Discussion

ALK TKIs have emerged as promising targeted therapy in ALK-driven neuroblastoma, although primary or acquired resistance to treatment occur frequently (13, 18–20, 24, 39, 40). We describe the effect of single-agent lorlatinib in five patients with *ALK*-mutated relapsed or refractory metastatic high-risk neuroblastoma. This is an unselected national cohort as it includes all patients with neuroblastoma treated with lorlatinib as monotherapy in Sweden. The median progression-free and overall survival have not been reached yet because three of the patients were still in remission at the latest follow-up but will be at least 35 and 55 months, respectively. These responses are notable compared with previous reports that show a median progression-free survival of 2 to 6 months and a median overall survival of 16 months in patients with relapsed or refractory neuroblastoma (41, 42).

Patients with *ALK* p.R1275Q have shown a trend toward better response to the first- and second-generation ALK inhibitors ceritinib and crizotinib compared with those with other *ALK* mutations (19, 39). The long-lasting responses seen in our cohort strengthen the rational for lorlatinib as monotherapy in patients with *ALK p.*R1275Q. Previous studies have also suggested that patients with germline *ALK* mutations respond well to ALK TKI treatment (18, 19, 24). This notion is further strengthened by the complete response seen in patient 3, who harbored a germline *ALK* p.R1275Q variant.

Goldsmith and colleagues (24) recently published data from a phase 1 clinical trial (NANT2015-02) with lorlatinib monotherapy in relapsed or refractory ALK-driven neuroblastoma, showing an overall response rate of 30% in children and 67% in adults. Very few responses were reported in patients with *MYCN* amplification, which is commonly seen in young children with high-risk neuroblastoma. In our study, three of the four patients with durable responses were over 11 years of age at the start of lorlatinib treatment, and none of them had an *MYCN* amplification. Together, these results raise the hypothesis that older patients with ALK-driven neuroblastoma may respond to lorlatinib as monotherapy, whereas young children harboring an *MYCN* amplification may benefit more from a combination regimen. The response rates of previous ALK TKI studies in patients with ALK-driven neuroblastoma are summarized in Supplementary Table S4.

We used a lorlatinib dose of 50 to 100 mg/m^2^, which was lower than the recommended phase 2 dose of 115 mg/m^2^ concluded in NANT2015-02. The main side effects of lorlatinib are known to be weight gain, hyperlipidemia, and affected neurocognitive functions (24), which were also seen in this cohort. However, all side effects could be managed with nutritional therapy, statin medication, and general support for the families, and were reversible after discontinuation of lorlatinib.

Monitoring ctDNA in liquid biopsies is an emerging method for evaluation of cancer treatment and detection of disease relapse. Previous studies have utilized whole-exome sequencing or broad NGS panels for ctDNA analysis in patients with neuroblastoma, which was focused on determining disease mechanisms rather than monitoring tumor burden (25, 29, 30). Alternatively, individualized assays have been developed for detection of one *ALK* mutation only, which may only be used in a minority of the patients (43). Here, we present a comprehensive NB-ALK sequencing panel that enables longitudinal ctDNA analysis in patients with ALK-driven relapsed or refractory neuroblastoma. Changes in ctDNA levels over time accurately reflected the clinical course of disease in all analyzed patients. In the two patients with relapse, ctDNA increased 9 and 3 months, respectively, before the appearance of any clinical symptoms, radiological findings, or elevated biochemical tumor markers. This increased sensitivity of relapsed disease detection offers a valuable window during which clinical treatment options can be considered.

One advantage of the NB-ALK sequencing panel is that the analysis is quantitative and may be used longitudinally to evaluate disease burden, not only for determining the presence or absence of mutations in ctDNA. The use of UMIs is critical as it allows for accurate quantification of DNA molecules in the original sample. Analyzing the levels of ctDNA with regular NGS panels that do not include UMIs is hampered by PCR or sequencing errors and uneven amplification, especially at low concentrations (44, 45).

A previous study by Berko and colleagues (25) made use of NGS panels covering 62 or 324 cancer-related genes for ctDNA analysis in patients with relapsed or refractory ALK-driven neuroblastoma treated with lorlatinib. Although suitable for detection of resistance mechanisms and powerful in the identification of novel mechanistic insight, broad NGS panels are not optimal for frequent analysis of measurable residual disease due to sequencing costs, limited sensitivity, and risk of false-positive results. Ultimately, a combination of the NB-ALK panel and a targeted NGS panel would achieve both sensitive monitoring of tumor burden and determination of resistance mechanisms. Patients can be monitored regularly with the NB-ALK panel during or after lorlatinib treatment and at the time of increased ctDNA levels indicating relapsed disease, and a broader NGS panel can be used to investigate the underlying mechanisms.

It is challenging to determine a relevant limit of detection for biomarker assays. Here, we report samples with at least one molecule harboring the mutation of interest (*ALK* p.R1275Q or p.F1174L) as ctDNA positive. The threshold was set low because of our use of UMIs that filter out PCR and sequencing errors. In patient 1, we detected one *ALK* p.R1275Q molecule in a sample 7 months after the end of treatment, whereas the other 11 follow-up samples were ctDNA negative. It remains uncertain whether the positive finding in this case was an accurate detection of the mutation or a technical error. In the clinical setting, low levels of ctDNA should be interpreted with caution and analysis of repeated samples may determine whether the finding is true or not.

Patient 5 was the only one in this cohort to experience disease progression during treatment with lorlatinib. Genetic analysis of the relapsed tumor performed as part of the clinical workup revealed an *HRAS* p.Q61L mutation that was previously shown to cause resistance to lorlatinib (46). We developed an *HRAS* sequencing assay based on similar methods as the NB-ALK panel and found that *HRAS* p.Q61L was detectable in circulating DNA only after 8 months of lorlatinib treatment. The simultaneous increase in allele frequency of *ALK* p.F1174L and *HRAS* p.Q61L leading up to the clinical relapse suggests that lorlatinib-resistant cells harbor both mutations. This is an example of how longitudinal ctDNA analysis can give insights about the genetic evolution of a tumor during oncologic treatment.

The frequency and completeness of standard response evaluations varied between the patients, limiting the possibility to comprehensively compare ctDNA results with clinically implemented methods for disease monitoring. We suggest ctDNA analysis to be performed once a month during and after lorlatinib treatment, although larger cohorts are needed to determine the optimal sampling interval.

In conclusion, we present an NB-ALK sequencing panel for monitoring disease at subclinical levels in patients with ALK-driven neuroblastoma. In combination with the current diagnostic modalities, ctDNA analysis offers a powerful tool to monitor treatment efficacy and inform clinical decisions during the treatment course, with the potential to improve clinical outcomes in patients with neuroblastoma. We also report major, long-lasting clinical responses to lorlatinib as monotherapy in five patients with ALK-driven relapsed or refractory neuroblastoma. These results are unexpected and underline the potential of targeted therapy in a group of patients with few other effective treatment options.

## Supplementary Material

Supplementary information Supplementary figures 1-4, supplementary tables 1-4, and supplementary case information

Figure S1 Sanger sequencing of tumor and germline DNA

Figure S2 Genomic profiles of tumor DNA generated by SNP microarray

Figure S3 Coverage with the NB-ALK sequencing panel

Figure S4 Levels of oncogenic ALK mutations in tissue samples

Supplementary data 1 SNP array results

Supplementary data 2 Sequencing results, all data

Supplementary data 3 Sequencing results, ALK hotspot mutation sites
