# Supplementary material for: Long-Lasting Response to Lorlatinib in Patients with ALK-Driven Relapsed or Refractory Neuroblastoma Monitored with Circulating Tumor DNA Analysis
Source: Cancer Res Commun. 2024 Sep 30;4(9):2553–64. doi: 10.1158/2767-9764.CRC-24-0338 (PMC11440348; doi:10.1158/2767-9764.CRC-24-0338)
Supplement: Supplementary information — Supplementary figures 1-4, supplementary tables 1-4, and supplementary case information [file crc-24-0338_supplementary_information_suppsi.docx]

**
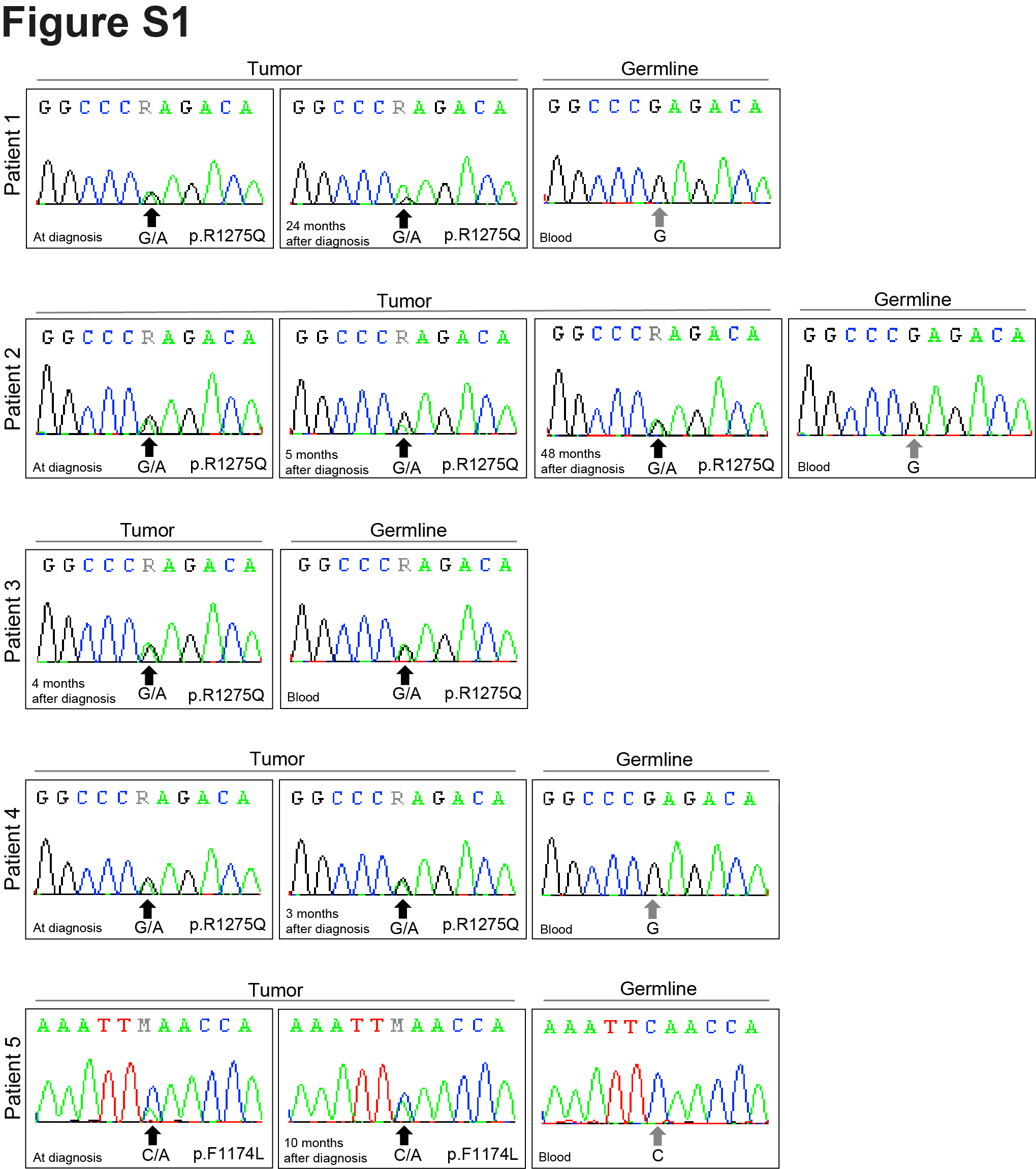
**

**Supplementary Figure 1.** Sanger sequencing of tumor and germline DNA. Electropherograms showing sequence covering the position corresponding to *ALK* R1275 or *F1174* in patient samples. Black and gray arrows show positions with and without *ALK* mutations, respectively.

**
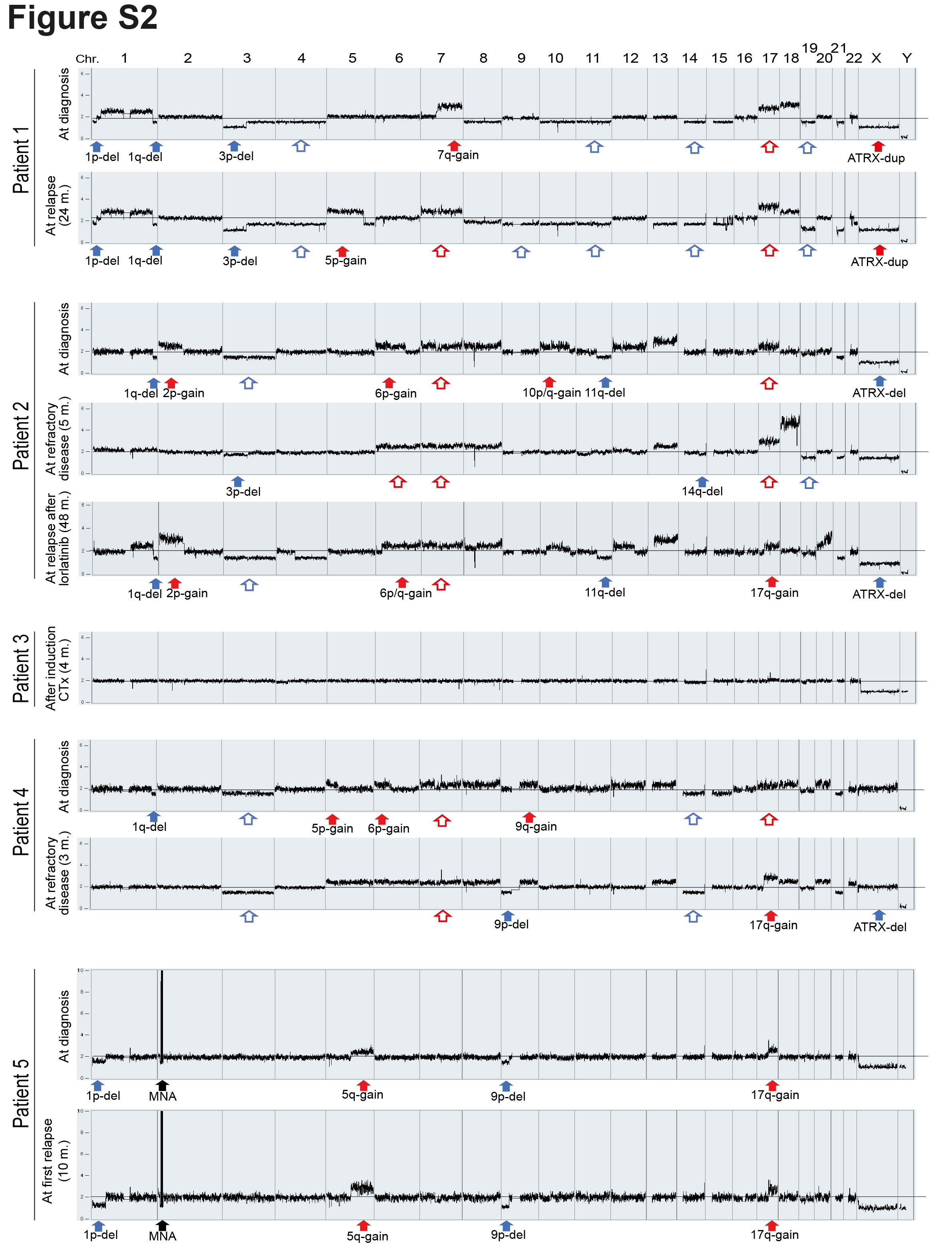
**

**Supplementary Figure 2**. Genomic profiles of tumor DNA generated by SNP microarray. Selected segmental aberrations are indicated in the genome-wide chromatogram plot as filled arrows (blue for deletion, red for gain and black for amplification). Open arrows show whole chromosome alterations. The modal karyotype of each sample is indicated with a horizontal line. Chr, chromosome; m, months after diagnosis; CTx, chemotherapy; del, deletion; dup, duplication; MNA, *MYCN* amplification.


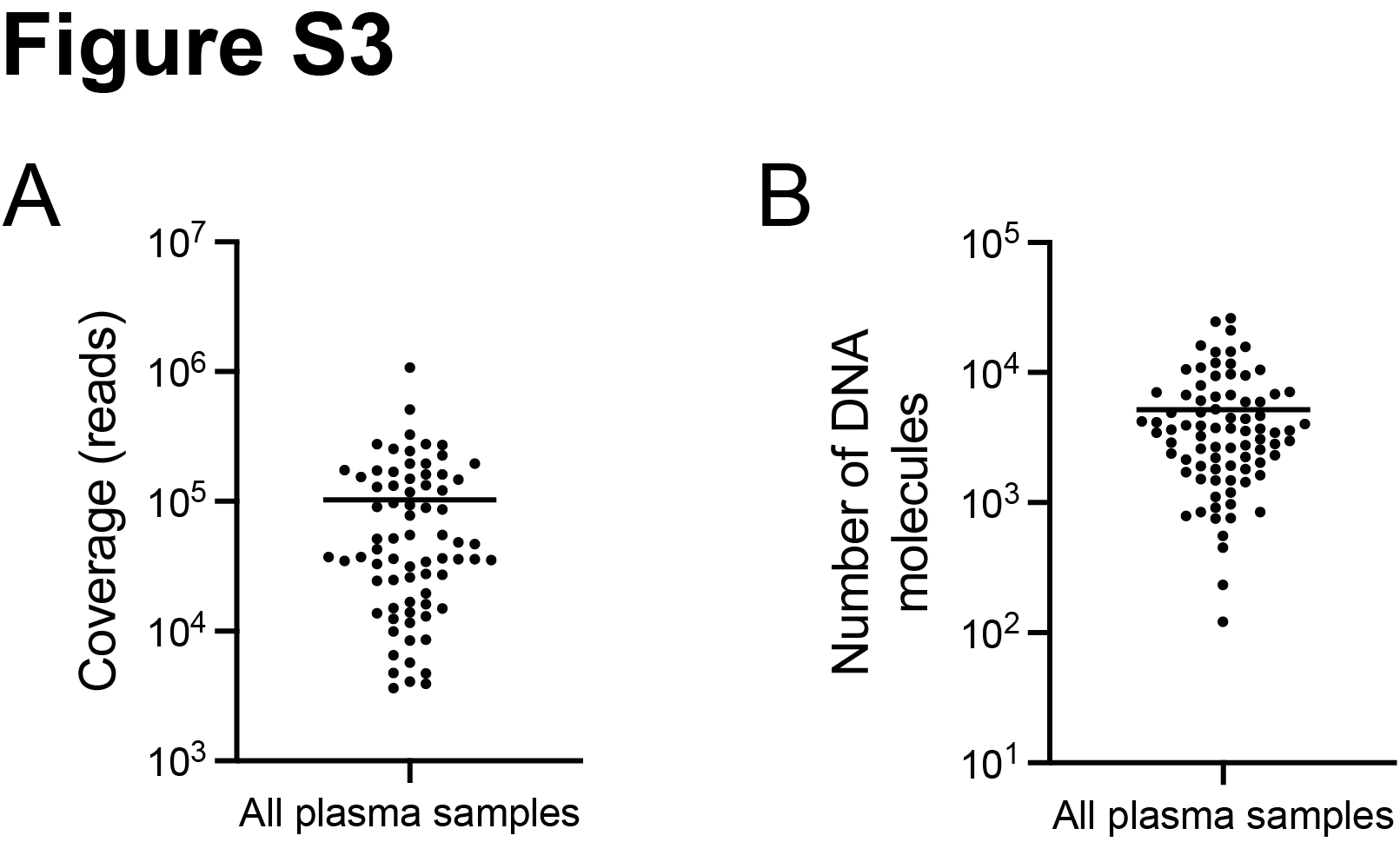


**Supplementary Figure 3.** Coverage with the NB-ALK sequencing panel. **A.** Average number of total reads per position of the panel, including only reads that had a UMI and were correctly aligned to the reference genome. B. Average number of DNA molecules at the site of the oncogenic mutation (*ALK* p.F1174 or p.R1275) that were sequenced at least three times with the same UMI. *N*=83 plasma samples.


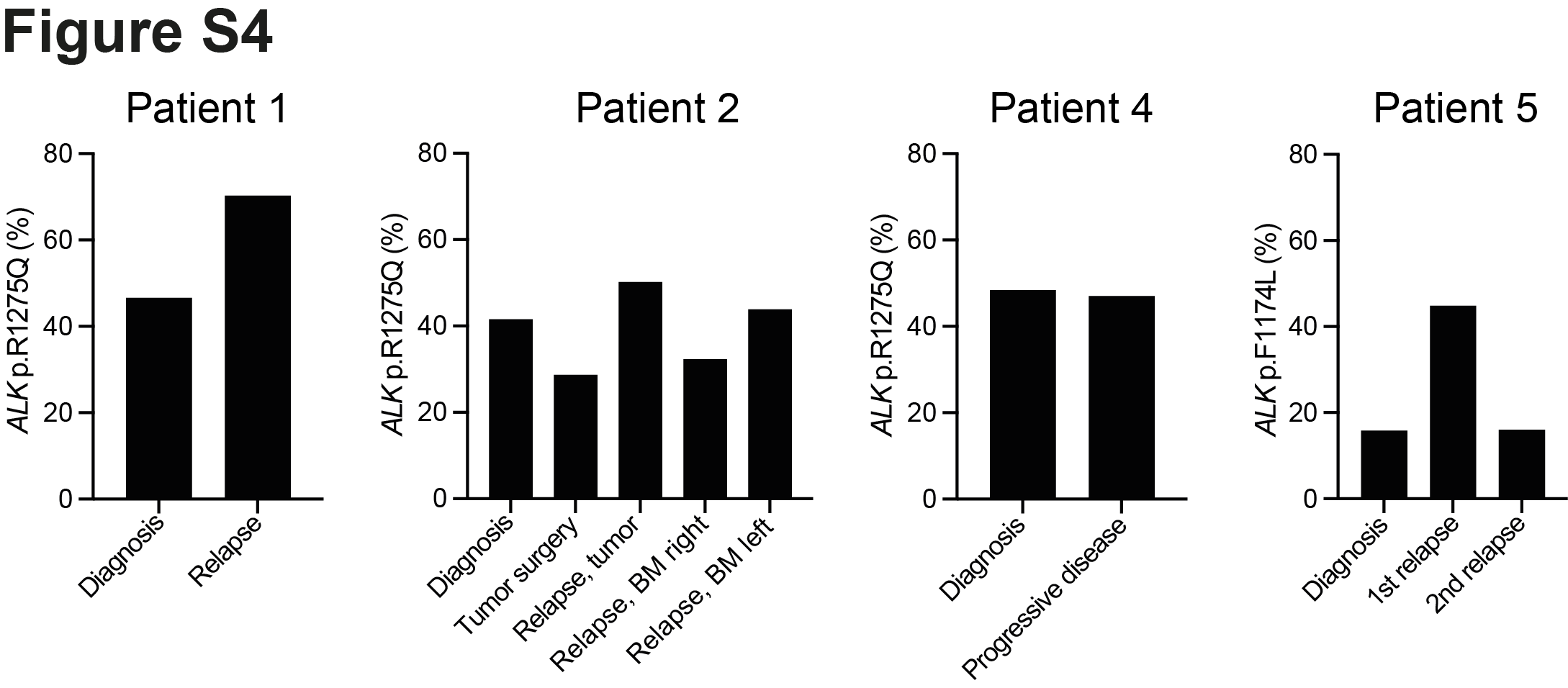


**Supplementary Figure 4**. Levels of oncogenic *ALK* mutations in tissue samples. BM, bone marrow.

| **Patient** | **Weight gain** | **Hypercholesterolemia** | **Peripheral edema** | **Neurocognitive symptoms** |
| --- | --- | --- | --- | --- |
| 1 | 1 | 2* | 1 | 1 |
| 2 | 3 | 1* | 1 | 0 |
| 3 | 3 | 1 | 1 | 2 |
| 4 | 2 | 1* | 1 | 1 |
| 5 | 1 | 1* | 0 | 0 |

**Supplementary Table 1**. Side effects during lorlatinib treatment. The symptoms are graded according to Common Terminology Criteria for Adverse Events (CTCAE) version 5.0, where 1 is mild and 5 is treatment-related death. *Treated with statins.

| **Assay^1^** | **Mutation** | **Nucleotide change** | **Nucleotide position** |
| --- | --- | --- | --- |
| 21 | p.L1122V | G>C | 29222603 |
|  | p.G1123S | G>A | 29222600 |
|  | p.G1128A | G>C | 29222584 |
| 22a | p.L1152R | T>G | 29222403-5 |
|  | p.C1156Y | G>A | 29222391-3 |
| 22b | p.I1170S | T>G | 29222350 |
|  | p.I1170N | T>A | 29222350 |
|  | p.I1171N | T>A | 29222347 |
|  | p.I1171T | T>C | 29222347 |
| 23a | p.F1174L | C>A | 29220829 |
|  | p.V1180L | G>C | 29220813 |
| 23b | p.L1196M | C>A | 29220765 |
|  | p.L1198F | C>T | 29220759 |
|  | p.G1202R | G>A | 29220747 |
|  | p.D1203N | G>A | 29220744 |
|  | p.L1204Y | C>G | 29220739 |
|  | p.E1210K | C>T | 29220723 |
| 24 | p.F1245V | T>G | 29213994 |
| 25 | p.G1269A | G>C | 29209816 |
|  | p.R1275Q | G>A | 29209798 |

**Supplementary Table 2**. Common primary and secondary *ALK* mutations covered by the NB-ALK panel.

^1^The assay is named after the exon number; two assays located in the same exon are named a and b.

| **Assay^1^** | ***ALK* amino acids^2^** | **Forward primer sequence** | **Reverse primer sequence** | **Amplicon size (bp)** |
| --- | --- | --- | --- | --- |
| 21 | 1120-1133 | TGTGGCCTGTTTGACTCTGTCTCCT | GGGCATTCCGGACACCTGGC | 104 |
| 22a | 1151-1160 | CCACCCTCCCCTTCTCTGCCC | GGGCTTCCATGAGGAAATCCAGTTCG | 78 |
| 22b | 1163-1172 | GGGCAGAGGGGAGTTGGGGT | TGTGCTCTGAACAGGACGAACTGG | 96 |
| 23a | 1172-1184 | TTGGTTACATCCCTCTCTGCTC | CAGGGATTGCAGGCTCACC | 82 |
| 23b | 1195-1214 | GGTTCTCACTCACCGGGC | TGCCCCGGTTCATCCT | 90 |
| 24 | 1238-1248 | GACATTGCCTGTGGCTGTC | AGCGACAGGATGACAGGAAG | 91 |
| 25 | 1268-1279 | CCCTGGAAGAGTGGCCAAG | GAGGGGTGAGGCAGTCTTTA | 79 |

**Supplementary Table 3**. Primers used for the NB-ALK panel.

^1^The assay is named after the exon number; two assays located in the same exon are named a and b. ^2^The range of ALK amino acid positions encoded by the amplicon target sequence. bp, basepairs.

| **Reference** | **N** | **Age (y)** | **ALK inhibitor** | **Mutations** | **Response rate** | **Complete response rate** | **Comment** |
| --- | --- | --- | --- | --- | --- | --- | --- |
| Goldsmith 2023 (1) | 23 | 1-18 | Lorlatinib  45-115 mg/m^2^ | p.F1174 48% p.F1245 24% p.R1275 24% | 13% | 4% | Cohort 1, dose-finding |
| Goldsmith 2023 (1) | 15 | >18 | Lorlatinib  100-150 mg | p.F1174 53% p.F1245 0% p.R1275 40% | 47% | 26% | Adults |
| Goldsmith 2023 (1) | 8 | <18 | Lorlatinib+  topotecan-cyclophosphamide | p.F1174 56% p.F1245 0% p.R1275 44% | 25% | 12% | Combination with chemotherapy |
| Stiefel 2023 (2) | 15 | 16-71 | Lorlatinib 87% 50-150 mg | p.F1174 53% p.R1275 27% other 20% | 60%  (69% lorlatinib) | 27% | Adults |
| Pastorino 2023 (3) | 8 | 4-22 | Crizotinib | p.F1174 75% p.R1275 12% p.S104R 12% | 20% | 20% | 3 combinations with chemotherapy |
| Fischer 2021(4) | 30 | 1-18 | Ceritinib 500 mg/m^2^ | ALK amp/trans-location 13%  p.F1174 17%  p.R1275 27%  p.F1245 7%  Other/unknown 40% | 20% | 0 |  |
| Foster 2021 (5) | 20 | 2-14 | Crizotinib | p.F1174 15% p.F1245 0% p.R1275 60% ALKamp 10% | 15% | 5% |  |
| Mossé 2013 (6) | 11 | 3-14 | Crizotinib | p.R1275 45% p.F1174 36% other 18% | 9% | 9% |  |
| *SUM* | *130* |  | *Lorlatinib 61*  *Ceritinib 30*  *Crizotinib 39* | | *25%* | *10%* |  |

**Supplementary Table 4.** Four clinical trials (Goldsmith, Fischer, Foster and Mossé) and two cohort studies (Stiefel and Pastorino) of neuroblastoma patients treated with ALK inhibitors.

**Supplementary case information**

*Patient 1*

The patient presented at nine years of age with multiple large retroperitoneal tumor components in the pancreas (~500 ml), the left adrenal gland (~10 ml) and multiple enlarged lymph nodes. Tumor markers were elevated, especially chromogranin A at 6.94 × upper reference limit (URL), neuron-specific enolase (NSE) at 9.57 × URL and methoxy-noradrenaline (mNA) at 5.82 × URL. Histopathological analysis of core needle biopsies confirmed a diagnosis of neuroblastoma. MIBG scan showed increased uptake at tumor sites but did not identify additional metastases. Analyses of bone marrow biopsies were negative. Array comparative genomic hybridization (CGH) of tumor biopsy identified multiple segmental chromosomal alterations (deletions of 1p, 1q, 3p and gain of 7q), but no amplification of *MYCN*, deletion of 11q or gain of 17q. Rapid COJEC induction treatment (SIOPEN HR-NBL protocol v 1.7) was started, and evaluation after eight courses showed a 65% reduction in tumor volume. The remaining tumor components were resected with macroscopic radicality. Autologous stem cell transplant (ASCT) with busulphan and melphalan conditioning was omitted due the complete remission status after surgery and no extra-abdominal metastasis. The patient received postoperative radiotherapy (21 Gy) followed by six cycles of 13-cis RA without anti-GD2 treatment.

Two years after initial diagnosis, chromogranin A levels increased to 4.2 × UNL. Computed tomography (CT) scan showed multiple thoracic metastases. Core needle biopsy verified a relapse of poorly differentiated neuroblastoma, and the *ALK* p.R1275Q mutation was confirmed with Sanger sequencing from tumor tissue. Bone marrow biopsies were negative, but MIBG scans showed increased uptake in the mediastinal tumor, os ileum, and in multiple locations of the lungs. Treatment with irinotecan and temozolomide was given and evaluation after two courses a showed minor regression (-25%) of the mediastinal lesion and the lung metastases. Treatment was changed to lorlatinib 75 mg/m^2^ once daily, with rapid and striking effects. Chromogranin A values normalized after three weeks and remained normal thereafter. Assessment with CT scan after eight weeks showed major regression of all thoracic components (mediastinal -90 %), and after six months complete regression of all tumor components, with a small thymic rebound in mediastinum. Subsequently, the lorlatinib dose was increased to 100 mg/m2. The observed side effects of lorlatinib were hypercholesterolemia grade 2 which was treated with rosuvastatin, weight gain (BMI +1 SD), mild peripheral edema and very mild neurocognitive affection.

After 40.6 months on lorlatinib with a complete remission according to the revised INRC-criteria, normal HVA, VMA and chromogranin A, and undetectable ctDNA, lorlatinib treatment was stopped. The patient is currently under careful monitoring including monthly ctDNA analysis using the NB-ALK panel.

*Patient 2*

The patient presented at 10 years of age with intermittent headache, chest pain and back pain for approximately six months. She also developed nutritional problems and weight loss, and finally diplopia and vomiting. Imaging revealed an occipital tumor mass, present both intra- and extracranially. MRI also identified multiple intraspinal tumors, expanding from Th2 to Th6, resulting in a significant compression of the spinal cord and vertebral body at Th4. Additionally, a left-sided adrenal gland tumor and radiological signs of bone marrow engagement were detected.

Cranial and spinal tumors were resected, and histopathology classified the tumor as a neuroblastoma. Genetic analyses revealed a somatic *ALK* p.R1275Q mutation and 11q-deletion. Whole genome sequencing (WGS) of peripheral blood showed two germline *FANCA* mutations of unknown significance.

Additional examination verified bone marrow engagement. Urine homovanillic acid (HVA), vanillylmandelic acid (VMA) and dopamine as well as NSE were elevated. MIBG showed general bone involvement (Curie score 20) and high uptake in the left adrenal gland. The patient was treated with Rapid COJEC according to SIOPEN HR-NBL 1.5 protocol with poor response followed by two pre-op courses Topotecan-Vincristine-Doxorubicin (TVD). After this, NSE, HVA and VMA were normalized and bone marrow biopsies were negative, but MIBG showed persistent bone uptake (Curie score 13). The patient underwent surgery with left adrenalectomy and resection of local lymph nodes. Tumor analysis indicated 40% viable tumor cells, and three out of seven lymph nodes showed tumor involvement. The patient received two postoperative TVD courses and continued treatment with high-dose Busulphan and Melphalan followed by autologous stem cell transplantation. Due to remaining MIBG uptake with Curie Score 8, the patient was taken off protocol and treated with lorlatinib at a dose of 75 mg/m^2^. After just a couple of weeks the patient developed respiratory symptoms leading to temporary suspension of lorlatinib for approximately one month during treatment of a potential respiratory infection. Lorlatinib was restarted with a 60% dose reduction and was increased to full dose after one month. Follow-up with MIBG which had shown partial regress of bone metastases (Curie Score 8) prior to start of lorlatinib treatment, indicated complete remission (Curie Score 0) after six months of treatment.

During lorlatinib treatment, the patient experienced weight gain (+2.5 SD, increasing in BMI from 17.3 to 24.9) as well as increased levels of triglycerides and cholesterol. Statin treatment was started but had to be discontinued due to intolerable side effects. The patient has subsequently received hormone substitution due to gonadal failure and hypothyroidism, which is likely resulting from the high-dose chemotherapy treatment.

After 32 months of lorlatinib, the patient remained in CR and the treatment was stopped. Nine months after discontinuing lorlatinib, a disseminated relapse occurred in bone (SIOPEN score 8) and bone marrow, as well as a minor soft tissue component in the pelvis. Serum VMA, NSE and CGA were markedly elevated. A core needle biopsy showed poorly differentiated neuroblastoma with high a proliferation rate (Ki-67 90%). Genetic analysis showed the *ALK* p.R1275Q mutation, 11q deletion, and 17q gain, but no obvious resistance mutation. Lorlatinib was reintroduced, leading to significantly improved general condition within three weeks. Evaluation after three months showed metabolic complete response on MIBG scan (SIOPEN score 0), stable disease on MRI, partial response in the bone marrow, and normalization of the biochemical tumor markers. After six months of lorlatinib monotherapy, the patient received consolidation treatment with temozolomide and irinotecan for five days and dinutuximab for seven days in cycles that were repeated every 21 days. Five such cycles were given during continuous lorlatinib treatment, and five additional cycles were given after discontinuation of lorlatinib. At the end of this treatment, the patient showed complete response on MRI, MIBG scan and bone marrow analysis.

*Patient 3*

The patient was diagnosed at two months of age with neuroblastoma in both adrenal glands and metastases in the bone marrow, liver, lung, brain, subcutis and multiple lymph nodes. MIBG showed high uptake in both adrenal tumors and metastases. Urine catecholamine metabolites were elevated at diagnosis. The initial diagnostic work-up was performed in another EU country and tissue samples were sent to Norway for the molecular analysis, which gave inconclusive results. Due to the widespread metastasis, the patient was stratified to high-risk treatment according to the SIOPEN HR-NBL 1 protocol. Two weeks after completion of Rapid COJEC the family moved to Sweden. Evaluation with MRI, MIBG, bilateral bone marrow aspiration and biopsy showed partial response of the primary tumors and complete response of metastases in the lungs, bones and dura, but there was still metastatic disease in the bone marrow and leptomeningeal carcinomatosis. Urine catecholamine metabolites and NSE were slightly elevated.

A biopsy from the primary tumor was taken and one course of chemotherapy with topotecan, vincristine, and doxorubicin (TVD) was given while waiting for the results. Molecular analysis showed a germline *ALK* p.R1275Q mutation without other significant genetic abnormalities. At that point the bone marrow was free from metastasis, urine catecholamine metabolites were still slightly elevated, and MRI as well as MIBG showed stable disease. The patient underwent stem cells harvest but did not receive high dose treatment.

Based on the genetic finding, treatment was started with lorlatinib at a dose of 90 mg/m^2^. After one month of treatment, urine catecholamine metabolites were normalized and evaluation with MRI and MIBG after five months showed substantial partial response. After seven months of treatment the patient underwent surgery of the adrenal tumors, and lorlatinib was paused for two weeks after the operation. Another evaluation after 12 months of lorlatinib showed complete response.

The patient experienced weight gain (from +0.5 SD to > +3 SD and a BMI increase from 15.3 to 24.5) and intermittently elevated cholesterol levels. Statin treatment was not given but cholesterol levels were monitored closely with blood samples at least once a month and regular nutritionist visits. At one point after 17 months of lorlatinib, the treatment was paused for three weeks due to the rapid and extensive weight gain. Other side effects included constipation which was treated with laxatives on a regular basis, and sleep disturbances. The patient was also diagnosed with developmental delay (motor skills and speech delay) and had frequent contact with the habilitation team during the lorlatinib treatment.

After almost three years of lorlatinib a new evaluation was made including MRI, MIBG, bilateral bone marrow biopsies, and urine catecholamine metabolites, showing that the patient remained in complete remission. The treatment was then discontinued, and the boy is currently monitored with clinical examination and biochemical check-up once a month and radiology and urine catecholamine metabolite analysis every three months. Two months after the end of treatment the body weight was dramatically reduced, and blood lipids were normalized. The parents then reported that the patient’s speech skills, ability to concentrate and stay calm, as well as the sleep were markedly improved compared to during lorlatinib treatment.

*Patient 4*

The patient presented at 18 years of age with acute spinal cord compression caused by a large retroperitoneal tumor with growth in Th12 and extension into the epidural space. Laminectomy and biopsy were performed, and histopathology indicated neuroblastoma. Fluorodeoxyglucose-positron emission tomography (FDG-PET) analysis identified skeletal metastases in sacrum and vertebrae Th11 and L3, as well as distant lymph node metastases. MIBG confirmed bone involvement and lymph node metastases (Curie score 6).

Bone biopsy from Th12 showed an unusual neural crest cell tumor. Tumor genetics identified an *ALK* p.R1275Q mutation. Copy number analysis showed a chromosome 1q deletion and gain of chromosome 5p, 6p and 9q, but no *MYCN* amplification. After initial treatment with Rapid COJEC, MIBG showed stable disease with Curie score 7. To achieve higher imaging resolution, a Ga-68-DOTATOC PET scan was also performed, which showed intense uptake in the tumor mass and unchanged multiple skeletal metastases. Debulking surgery of the retroperitoneal tumor was performed, and histopathological analysis identified a ganglioneuroblastoma with highly malignant components and signs of differentiation. Re-evaluation after two courses of TVD showed progressive disease. Autologous stem cell transplantation with busulfan and melphalan was cancelled, and the patient was switched to lorlatinib monotherapy. The starting dose was 28 mg/m^2^, which was increased to 56 mg/m^2^ after seven weeks of treatment.

A rapid metabolic response was noted with normalization of methoxy-noradrenaline after three weeks of lorlatinib treatment. Patient reevaluation with Ga-68-DOTATOC PET after five and nine months on lorlatinib indicated a complete metabolic response both in the tumor mass and all skeletal metastases. CT scan after 5, 9 and 29 months showed the continued presence of a cystic, retroperitoneal tumor, but no new lesions were identified. The patient remains on lorlatinib after 35 months of treatment, with hypercholesterolemia grade 1 as only side effect.

*Patient 5*

The patient presented at 22 months of age with poor general condition, fever and a tumor in the right mandible. A full evaluation identified a tumor in the right adrenal gland, as well as multiple metastases in the lungs, liver, bone and bone marrow. Genetic analysis of the tumor identified *MYCN* amplification, chromosome 1p deletion, chromosome 17q gain, and an *ALK* p.F1174L mutation. Induction chemotherapy with Rapid COJEC resulted in metastatic complete response, and the adrenal tumor was resected. The patient was then treated with autologous stem cell transplantation with busulfan and melphalan, local radiotherapy with 21 Gray to the tumor bed, and maintenance treatment with 13-cis-retinoic acid and dinutuximab beta. After two of five maintenance cycles a metastatic relapse occurred in the mandible. Genomic analysis of biopsy material confirmed *MYCN* amplification and the *ALK* *F1174L* mutation with a variant allele frequency (VAF) of 36%. MIBG investigation showed a single hot-spot in the mandible (SIOPEN score 1) and the bone marrow biopsy was free of tumor cells. One course of TVD was initiated immediately, followed by one course of topotecan and cyclophosphamide (TC) and concomitant initiation of lorlatinib (98 mg/m^2^). After three months of lorlatinib monotherapy a CT scan showed partial regression of the mandibular tumor and after 4.5 months, a new MIBG investigation indicated a decreased uptake in the mandible, but a weak uptake in the right fibula that could not be detected with CT.

The main side effects of lorlatinib were hypercholesterolemia grade 1 which was controlled by rosuvastatin, and weight gain (BMI +1 SD). After 10.5 months on lorlatinib treatment, a second relapse occurred in the same mandibular region as the first relapse. The biopsy was examined with the next-generation sequencing panel Oncomine Focus, which showed *MYCN* amplification, the *ALK* p.F1174L mutation (VAF 30%) and an *HRAS* p.Q61L mutation (VAF 32%) that had not been present in the first relapse biopsy. No resistance mutations were found in the *ALK* gene. The lorlatinib treatment was stopped, since the resistance was presumed to be caused by the activation of the RAS-MAPK pathway. Treatment with TEMIRI and bevacizumab stabilized the tumor for four months. An attempt to add dinutuximab failed and the patient succumbed to progressive disease.

**References**

1. Goldsmith KC, Park JR, Kayser K, Malvar J, Chi YY, Groshen SG, et al. Lorlatinib with or without chemotherapy in ALK-driven refractory/relapsed neuroblastoma: phase 1 trial results. Nat Med. 2023;29(5):1092-102.

2. Stiefel J, Kushner BH, Roberts SS, Iglesias-Cardenas F, Kramer K, Modak S. Anaplastic Lymphoma Kinase Inhibitors for Therapy of Neuroblastoma in Adults. JCO precision oncology. 2023;7:e2300138.

3. Pastorino F, Capasso M, Brignole C, Lasorsa VA, Bensa V, Perri P, et al. Therapeutic Targeting of ALK in Neuroblastoma: Experience of Italian Precision Medicine in Pediatric Oncology. Cancers. 2023;15(3).

4. Fischer M, Moreno L, Ziegler DS, Marshall LV, Zwaan CM, Irwin MS, et al. Ceritinib in paediatric patients with anaplastic lymphoma kinase-positive malignancies: an open-label, multicentre, phase 1, dose-escalation and dose-expansion study. Lancet Oncol. 2021;22(12):1764-76.

5. Foster JH, Voss SD, Hall DC, Minard CG, Balis FM, Wilner K, et al. Activity of Crizotinib in Patients with ALK-Aberrant Relapsed/Refractory Neuroblastoma: A Children's Oncology Group Study (ADVL0912). Clinical cancer research : an official journal of the American Association for Cancer Research. 2021;27(13):3543-8.

6. Mossé YP, Lim MS, Voss SD, Wilner K, Ruffner K, Laliberte J, et al. Safety and activity of crizotinib for paediatric patients with refractory solid tumours or anaplastic large-cell lymphoma: a Children's Oncology Group phase 1 consortium study. Lancet Oncol. 2013;14(6):472-80.
