# Supplementary material for: Long-Lasting Response to Lorlatinib in Patients with ALK-Driven Relapsed or Refractory Neuroblastoma Monitored with Circulating Tumor DNA Analysis
Source: Cancer Res Commun. 2024 Sep 30;4(9):2553–64. doi: 10.1158/2767-9764.CRC-24-0338 (PMC11440348; doi:10.1158/2767-9764.CRC-24-0338)
Supplement: Figure S1 — Sanger sequencing of tumor and germline DNA [file crc-24-0338_figure_s1_suppsf1.docx]

**
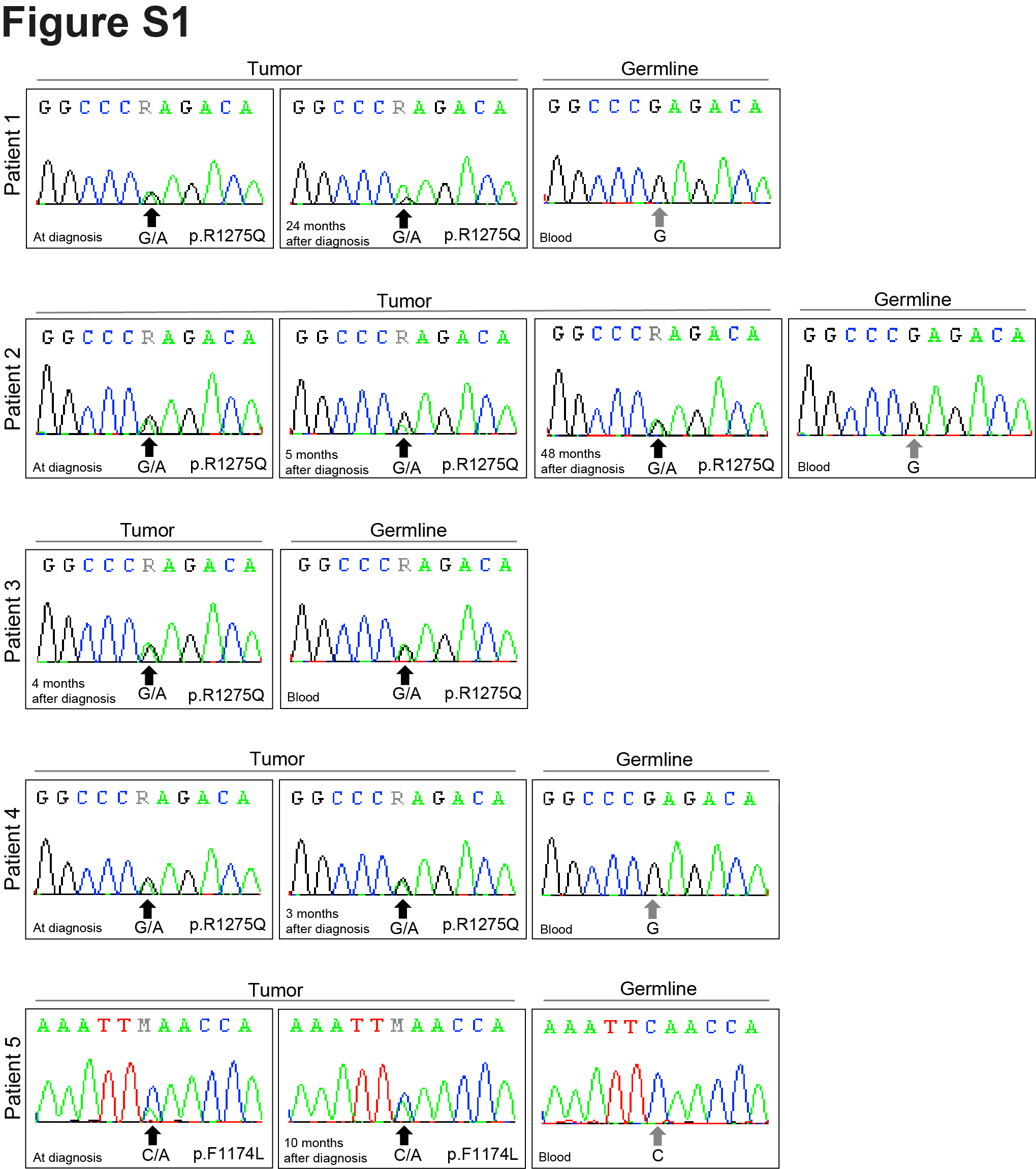
**

**Supplementary Figure 1.** Sanger sequencing of tumor and germline DNA. Electropherograms showing sequence covering the position corresponding to *ALK* p.R1275 or p.F1174 in patient samples. Black and gray arrows show positions with and without *ALK* mutations, respectively.
