# Supplementary material for: Long-Lasting Response to Lorlatinib in Patients with ALK-Driven Relapsed or Refractory Neuroblastoma Monitored with Circulating Tumor DNA Analysis
Source: Cancer Res Commun. 2024 Sep 30;4(9):2553–64. doi: 10.1158/2767-9764.CRC-24-0338 (PMC11440348; doi:10.1158/2767-9764.CRC-24-0338)
Supplement: Figure S2 — Genomic profiles of tumor DNA generated by SNP microarray [file crc-24-0338_figure_s2_suppsf2.docx]

**
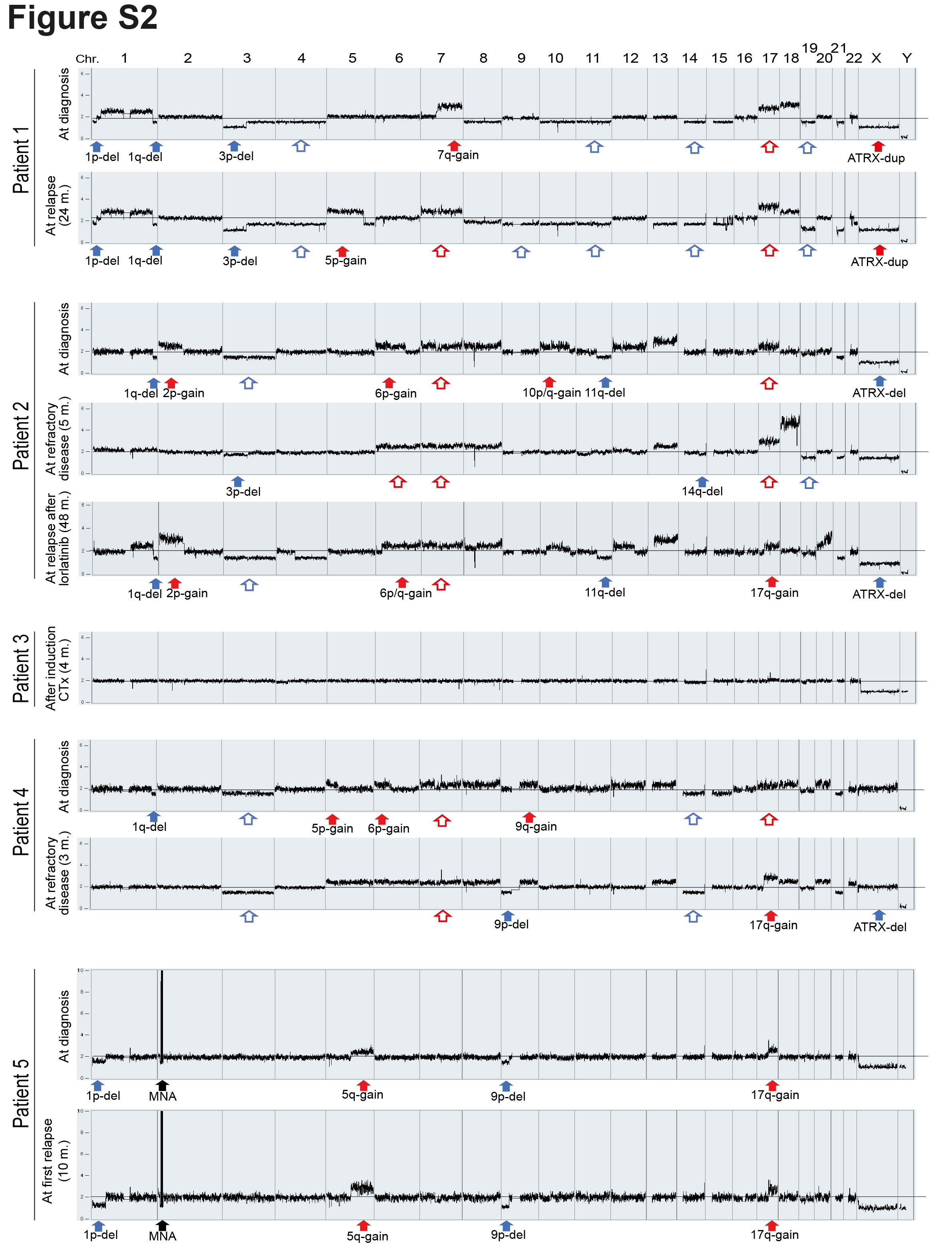
**

**Supplementary Figure 2**. Genomic profiles of tumor DNA generated by SNP microarray. Selected segmental aberrations are indicated in the genome-wide chromatogram plot as filled arrows (blue for deletion, red for gain and black for amplification). Open arrows show whole chromosome alterations. The modal karyotype of each sample is indicated with a horizontal line. Chr, chromosome; m, months after diagnosis; CTx, chemotherapy; del, deletion; dup, duplication; MNA, *MYCN* amplification.
