# Supplementary material for: Long-Lasting Response to Lorlatinib in Patients with ALK-Driven Relapsed or Refractory Neuroblastoma Monitored with Circulating Tumor DNA Analysis
Source: Cancer Res Commun. 2024 Sep 30;4(9):2553–64. doi: 10.1158/2767-9764.CRC-24-0338 (PMC11440348; doi:10.1158/2767-9764.CRC-24-0338)
Supplement: Figure S3 — Coverage with the NB-ALK sequencing panel [file crc-24-0338_figure_s3_suppsf3.docx]

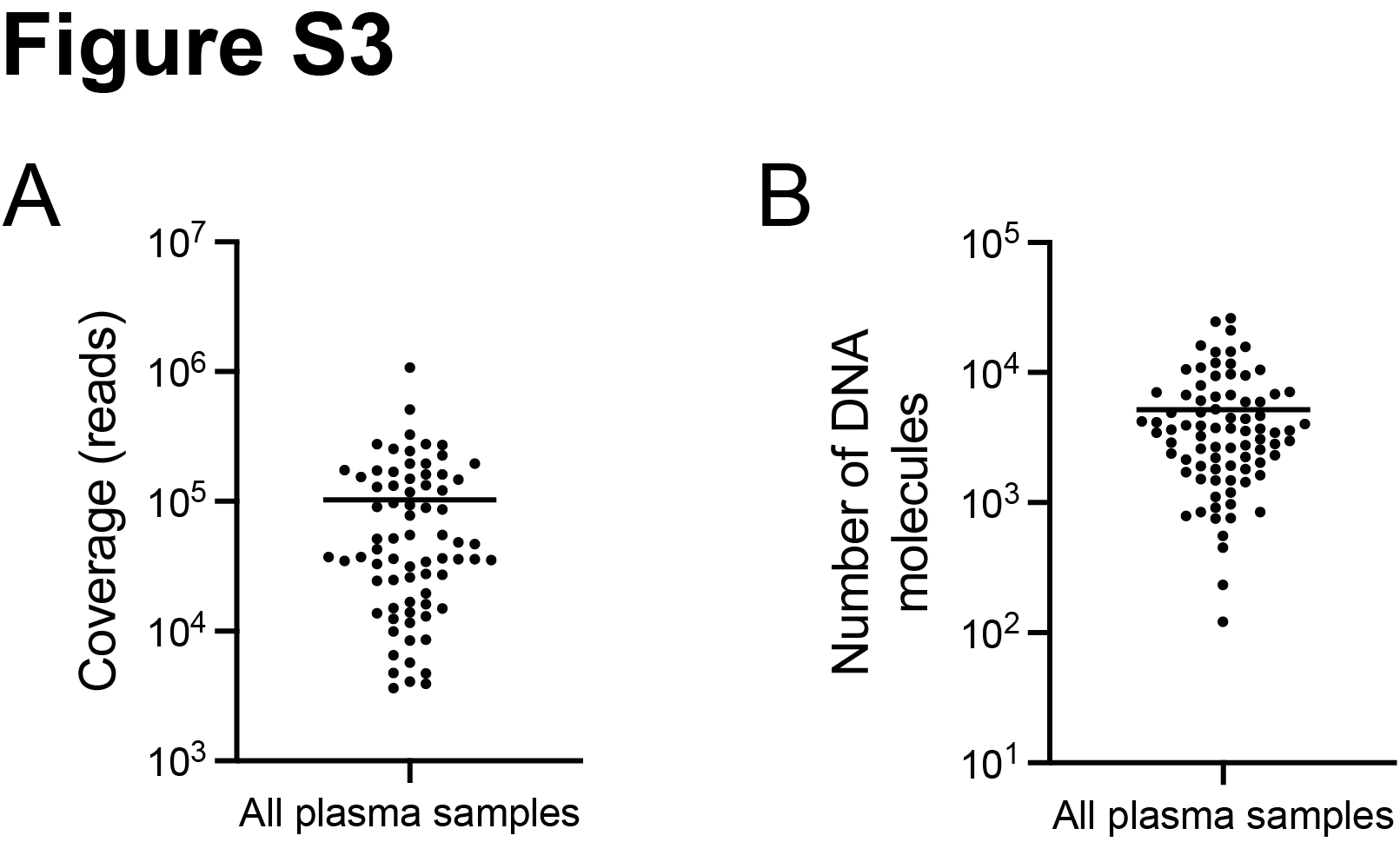


**Supplementary Figure 3.** Coverage with the NB-ALK sequencing panel. **A.** Average number of total reads per position of the panel, including only reads that had a UMI and were correctly aligned to the reference genome. **B.** Average number of DNA molecules at the site of the oncogenic mutation (*ALK* p.F1174 or p.R1275) that were sequenced at least three times with the same UMI. *N*=83 plasma samples.
