# Supplementary figures and images for: Long-Lasting Response to Lorlatinib in Patients with ALK-Driven Relapsed or Refractory Neuroblastoma Monitored with Circulating Tumor DNA Analysis
Source: Cancer Res Commun. 2024 Sep 30;4(9):2553–64. doi: 10.1158/2767-9764.CRC-24-0338 (PMC11440348; doi:10.1158/2767-9764.CRC-24-0338)

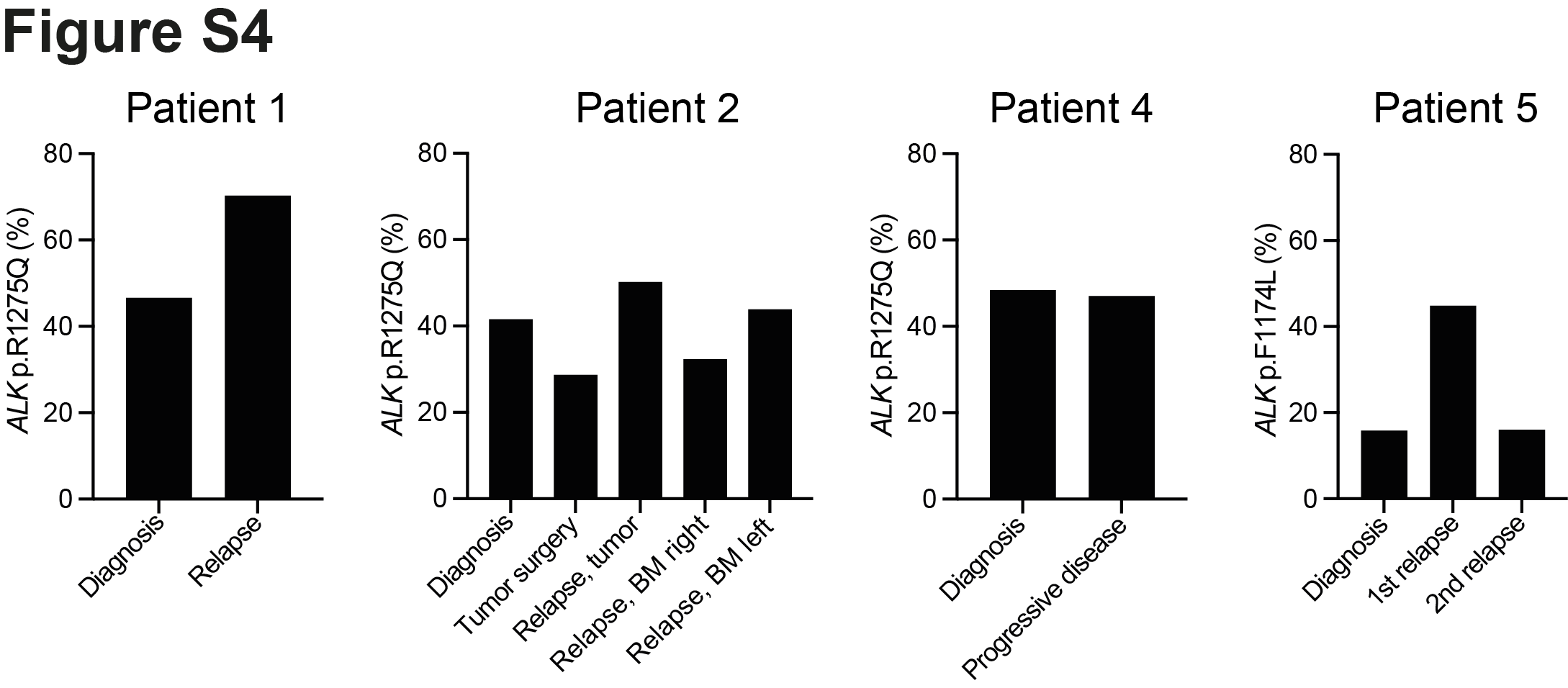


**Supplementary Figure 4**. Levels of oncogenic *ALK* mutations in tissue samples. BM, bone marrow.

Supplement: Figure S4 — Levels of oncogenic ALK mutations in tissue samples [file crc-24-0338_figure_s4_suppsf4.docx]
